# Supplementary figures and images for: Deletion of Oncomodulin Gives Rise to Early Progressive Cochlear Dysfunction in C57 and CBA Mice
Source: Front Aging Neurosci. 2021 Nov 15;13:749729. doi: 10.3389/fnagi.2021.749729 (PMC8634891; doi:10.3389/fnagi.2021.749729)

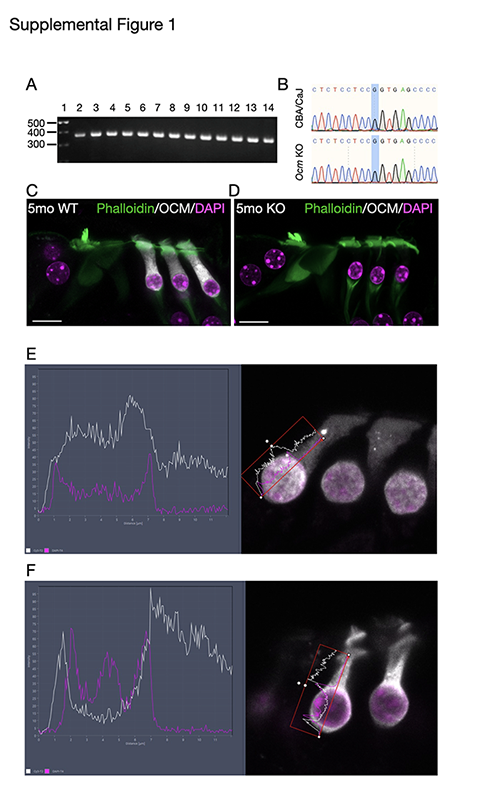

Supplement: Supplementary Figure 1 — (A) 360 bp PCR product for 12 CBA Ocm KO animals (lanes 2-13) and 1 CBA/CaJ control (lane 14). The DNA ladder is shown in lane 1. (B) Chromatograph of sequencing results from lane 2 and lane 14 showing that neither KO nor control animals have the G- > A Cdh23ahl mutation. (C,D) 5 mo old CBA wild type (WT) (C) and CBA Ocm KO (D) mid-modiolar sections stained with antibodies to Oncomodulin (OCM) (gray). Co-stained with phalloidin (green) and DAPI (magenta). Scale bar = 10 μm. (E,F) Line profile of 1 mo old (E) and 20 mo old (F) CBA WT mid-modiolar section stained with antibodies to OCM (gray) and co-stained with DAPI (magenta). [file Image_1.TIFF]

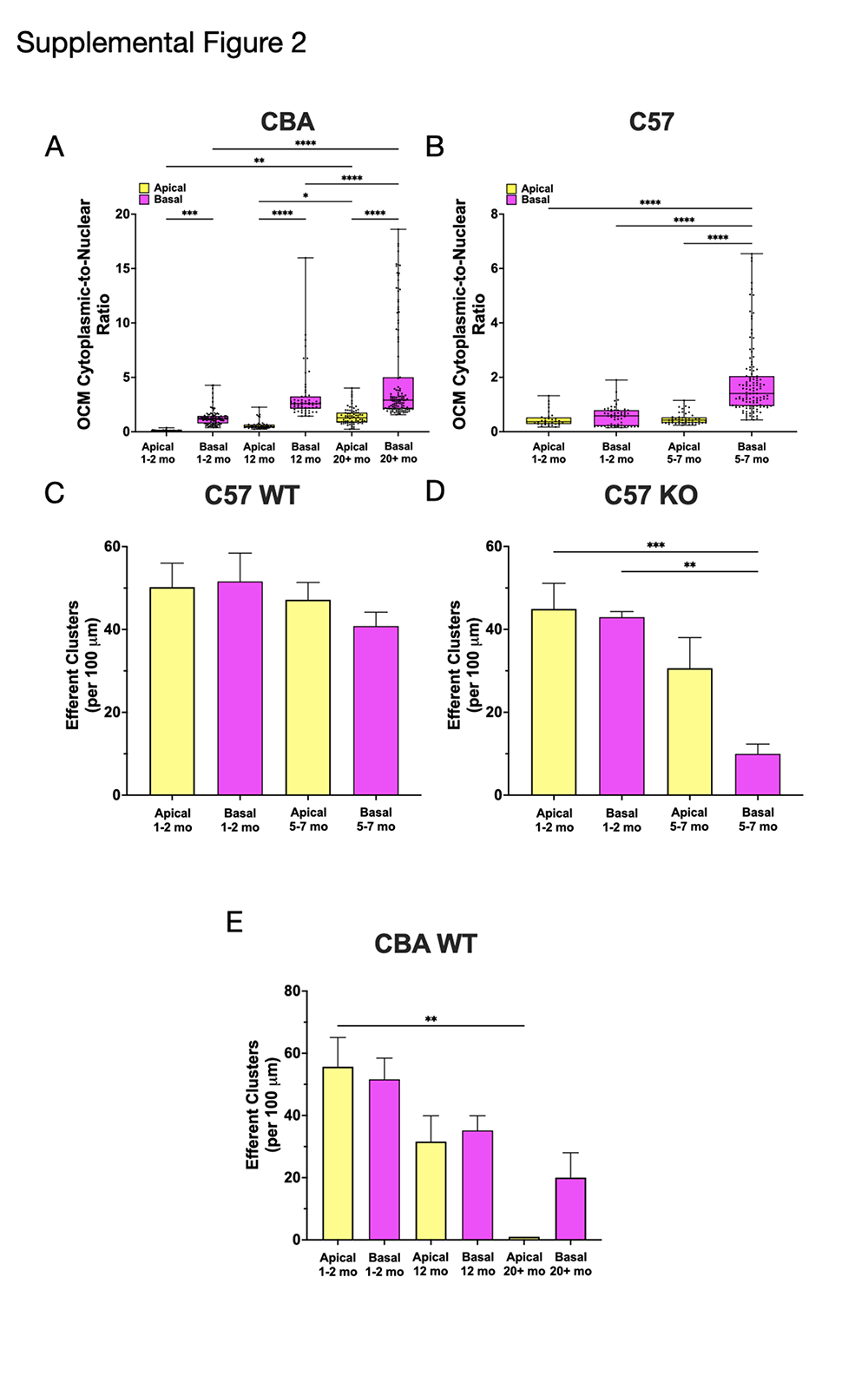

Supplement: Supplementary Figure 2 — Cytoplasmic vs. nuclear OCM fluorescence during aging in WT (A) CBA and (B) C57 mice. (A) 8 and 32 kHz c/n ratios from region of interests (ROIs) at 1 – 2 mo, 12 mo, and 20-30 mo (n = 5) CBA WT mice. (B) 8 kHz and 22 kHz c/n ratios from ROIs at 1 – 2 mo (n = 2) and 5 – 7 mo (n = 2) in C57 WT mice. Quantification of choline acetyltransferase (ChAT) labeled efferent clusters in the 5 – 8 kHz and 22 – 40 kHz regions of panels (C) C57 WT and (D) C57 KO. No statistical difference in ChAT clusters between any C57 WT regions. All WT regions were statistically significant (p < 0.5) from basal region of 5 – 7 mo KO. (E) ChAT clusters from the 8 and 22 - 45 kHz regions of WT CBA mice at 1 – 2 mo, 12 mo (n = 2) and 28 mo (n = 2). 3 cochlea per condition unless otherwise indicated. All asterisks indicate p < 0.5. [file Image_2.TIFF]
